# Based on metabolomics, the optimum wind speed process parameters of flue-cured tobacco in heat pump bulk curing barn were explored

**DOI:** 10.1038/s41598-023-49020-5

**Published:** 2023-12-06

**Authors:** Cheng Lin Sun, Hui Lin Zhang, Dong Bo Zhou, Zhi Jun Cheng, You Xie, Zhong Wen Rang, Lin Jian Dai

**Affiliations:** 1 College of Agriculture, Hunan Agricultural University, Changsha, 410128 China; 2 Raw Material Procurement Center, China Tobacco Hunan Industrial Co., Ltd., Changsha, 410000 China; 3 Technical Centre, China Tobacco Hunan Industrial Co., Ltd, Changsha, 410000 China; 4 Tongren City Company Jiangkou Branch, China Tobacco Corporation Guizhou Province Company, Tongren, 554300 China

**Keywords:** Plant sciences, Engineering

## Abstract

To explore the influence of wind speed on the quality of tobacco in this study, we employed a heat pump-powered intensive curing barn and a three-stage curing process. By evaluating the influence of fan parameters on the quality of tobacco leaves at different curing stages, the optimal wind speed was determined. After adopting the optimized wind speed process, the degradation of macromolecular substances was faster, the accumulation of aroma substances was delayed to 55 °C, and the accumulation was more complete. Among them, the contents of reducing sugar and total sugar in flue-cured tobacco leaves were 22.25% and 29.2%, respectively, which were lower than those in the control group. The sugar was converted into more aroma substances, and the total amount of neutral aroma substances was 48.82% higher than that of the control group. The content of related aroma substances increased significantly. The content of petroleum ether extract related to aroma substances increased by 0.93% compared with the control group. The macromolecular substances were degraded more fully than the control group, such as the starch content decreased to 1.56%. The results of metabolomics showed that the contents of aldehydes, heterocyclic compounds, alcohols, ketones and esters increased significantly in different degrees after this process. These results show that the optimization of wind speed parameters can significantly improve the baking quality of tobacco leaves. This study provides a reference for the optimization of the flue-cured tobacco baking process.

## Introduction

Baking is the most important part of flue-cured tobacco production and directly affects the quality of flue-cured tobacco^1–3^. With the large-scale use of bulk curing barns in flue-cured tobacco production, the amount of tobacco in the curing barn is further increased, but this method is accompanied by problems such as uneven distribution of temperature and humidity in the curing barn and large variations in wind speed between leaves, which causes the quality of tobacco leaves in the same curing barn to be significantly different^4,5^. To address these problems regarding bulk curing barns, many possible solutions have been explored. The temperature and humidity environment within the curing barn are the direct factors determining the chemical quality of tobacco leaves^6,7^. To a certain extent, different temperature and humidity conditions strengthen or inhibit the degradation of macromolecular substances and the synthesis of aroma substances in tobacco leaves^8,9^. Macromolecular substances such as starch are converted into precursors of aroma substances under suitable temperature and humidity conditions, such as small molecular sugars^10,11^. Research has found that temperature and humidity conditions that affect the quality of tobacco leaves mainly act on the regulation of the water loss rate in tobacco leaves^12,13^. The change in water content directly affects the key chemical reactions and enzyme activities in tobacco leaves^14–16^. The reasonable loss of water is the key factor that determines the quality of flue-cured tobacco^17,18^. Previous studies have been relatively complete, but previous studies have ignored the factors that play an indirect role between temperature, humidity conditions and tobacco leaves during the baking process-wind. Air, as a heat carrier, transfers heat to the leaves, and at the same time, as a moisture carrier, removes water from the leaves^19–21^. The speed of the fan determines the air flow rate, affects the heat transfer and temperature and humidity regulation inside the curing barn, plays a direct role in the water loss rate of the tobacco leaves, and plays an indirect role in the material changes inside the tobacco leaves. Wind speed plays an important role in the production of flue-cured tobacco and is an important part of the baking process^22,23^. Therefore, in this study, the wind speed utilized in the production of flue-cured tobacco was taken as the main research object. A heat pump bulk curing barn was used as the curing equipment to study the accumulation and variation of aroma substances in different curing stages and the influence of various fan speeds on the tobacco leaves. The appropriate fan parameters were established, and the verification test was assembled. The composition of flavor metabolites under this process was explored by metabolomics technology. At the same time, the changes in chemical reactions and main chemical components in the process of tobacco aroma formation were considered to provide a reference for improving the tobacco curing process.

## Materials and methods

### Test site and materials

The experiment was carried out in the bulk curing barn group in Guiyang County (HY:112.72° E, 25.73° N, Heigh: 302 m) , Chenzhou City, Hunan Province, in July 2022, and the tested flue-cured tobacco variety was Yunyan 116. During the experiment, the temperature change was small ( 30–32 °C ), and there was no rainfall. The tobacco leaves to be studied were the middle leaves with basically the same maturity. The heat pump system of Hunan Xindi Company's flue-cured tobacco room was used in three bulk curing barns, and a Fujian Huada variable frequency fan (Hander-A V10) was used to vary the wind speed.

### Test method

#### Experimental design

In this experiment, three groups, medium wind speed (MWS), low wind speed (LWS) and high wind speed (HWS) were established. Different wind speeds were established at each stage of baking, and the fan parameters suitable for different stages were screened according to the change trend of neutral aroma content in tobacco leaves. By measuring the internal wind speed of the bulk curing barn under different fan parameters, the corresponding data were obtained (Table 1). The fan parameters of the current bulk curing barn design are only high and low, and the wind speed is measured to be equivalent to 900 r/min and 1300 r/min. Because the yellowing stage of tobacco leaves is the key period for the accumulation of tobacco precursors, and the control requirements for the water loss rate of tobacco leaves are high in the late yellowing stage^24,25^, in this experiment we divided the yellowing stage into two stages with 38 °C as the boundary. Based on this, the fan speed for different stages was established (Table 2).

**Table 1:** Internal wind speed of curing barn under different fan speed.

Fan speed(r/min)	500	600	700	800	900	1000	1100	1200	1300	1400
Wind velocity(m/s)	3.56	3.99	4.38	4.77	5.20	5.59	5.95	6.37	6.73	7.11

**Table 2:** Fan speed parameters at different stages.

	Baking-38 °C	38–42 °C	42–48 °C	48–55 °C	55–65 °C
MWS	800 r/min	850 r/min	1350 r/min	900 r/min	750 r/min
LWS	750r/min	800 r/min	1300 r/min	850 r/min	700 r/min
HWS	850r/min	900 r/min	1400 r/min	950 r/min	800 r/min

#### Verification test

A three-stage baking process was employed. After screening the parameters of the fan in each stage of baking, a verification test was carried out, and two groups were established. One group used the variable frequency fan, and the selected fan parameters were recorded as T_1_; the other group used the current two-speed fan according to the wind speed change of the three-stage baking process (low-speed fan was used in the yellowing period, high-speed fan was used in the color fixing period, and low-speed fan was used in the drying period)^26,27^, recorded as CK.

### Sampling method

Before each treatment, 18 pieces of tobacco leaves in the upper, middle and lower layers of the curing barn were randomly selected and marked^28^. During the curing process, one-sixth of the marked tobacco leaves were evenly distributed and punched, and 6 pieces of tobacco leaves in the upper, middle and lower layers were mixed into one group, for a total of three groups. The key temperature points (dry bulb temperature: 38 °C, 42 °C, 48 °C, 55 °C, 65 °C) were sampled before and during the curing process.

### Determination items and methods

#### Neutral aromatic substances

Analysis was performed using a gas chromatography-mass spectrometer. Each sample was mixed in equal amounts to make a quality control ( QC ) sample, and one QC sample was inserted into each of the six test analysis samples to investigate the repeatability of the analysis process^29^. For determination of samples, the GC conditions were as follows: DB-5 elastic quartz capillary column, 60 m × 0.25 mm × 0.25 μm; injector temperature: 270 °C; split ratio: 20: 1, injection volume: 1 μl; carrier gas: helium, constant current 1 mL/min; programmed temperature: 60 °C (hold 0.5 min) 2 °C/min 280 °C (hold 30 min). MS conditions : transmission line temperature : 280 °C ; ion source temperature : 230 °C ; ionization mode : EI ; ionization voltage : 70 eV ; mass range : 30–350 ; solvent delay : 6 min.

#### Metabolomics

The metabolites were analyzed by GC112A gas chromatography and SCIEX mass spectrometry^29^. A DB-5MS capillary column (30 m × 250 μm i.d., 0.25 unfilm thickness) was used for GC‒MS separation with a constant current of 1 m L/min helium. A split ratio of 1:10 was used to inject 1 μL. The inlet temperature was 280 °C, the transmission line temperature was 320 °C, and the ion source temperature was 230 °C. Temperature programming was as follows: The initial temperature was maintained at 50 °C for 0.5 min and then increased to 320 °C at a rate of 15 °C/min and maintained for 9 min. Full scan mode was used, the scan rate was 10 spec/s, the electron energy was -70 V, and the solvent delay was 3 min.

#### Petroleum ether extract

Extraction of petroleum ether extract using soxhlet extractor^30^.15 g of flue-cured tobacco powder sample was extracted by Soxhlet extractor, 100 mL of petroleum ether ( 60 ~ 90 °C ) was added, refluxed for 2 h, and filtered. The tobacco residue was added with 100 mL petroleum ether ( 60 ~ 90 °C ), refluxed for 1.5 h, filtered, combined with filtrate, concentrated under reduced pressure, and dried to obtain petroleum ether extract.

#### Main chemical constituents.

The contents of the main chemical components in flue-cured tobacco leaves were determined by a flame photometer and continuous flow injection analyzer. The determination items and standards were as follows: starch: YC/T 216–2007; 2011; water-soluble sugar: YC/T 159–2002.

### Data statistics and analysis

Office 2019 was used to preliminarily classify and process the data. A completely randomized experimental design was used with 3 replicates per treatment. Statistical analysis was performed using analysis of variance^31^. Through IBM SPSS Statistics 25 software, independent sample T test, Pearson correlation test and other methods were used to test the significance between each group of data^32^ . After testing and classifying the data, GraphPad Prism8 is used to draw and layout the pictures. In the chart, the error line of each set of data is calculated and added by default by GraphPad Prism8. All the pictures in the article are done using GraphPad Prism8, and use it to combine small graphs into a group graph.

## Results and analysis

### Screening of wind speed in each stage of baking

The wind speed at different stages of the curing process was adjusted, and the appropriate fan parameters were selected in turn according to the changes in neutral aroma content in tobacco leaves.

**Figure 1:**
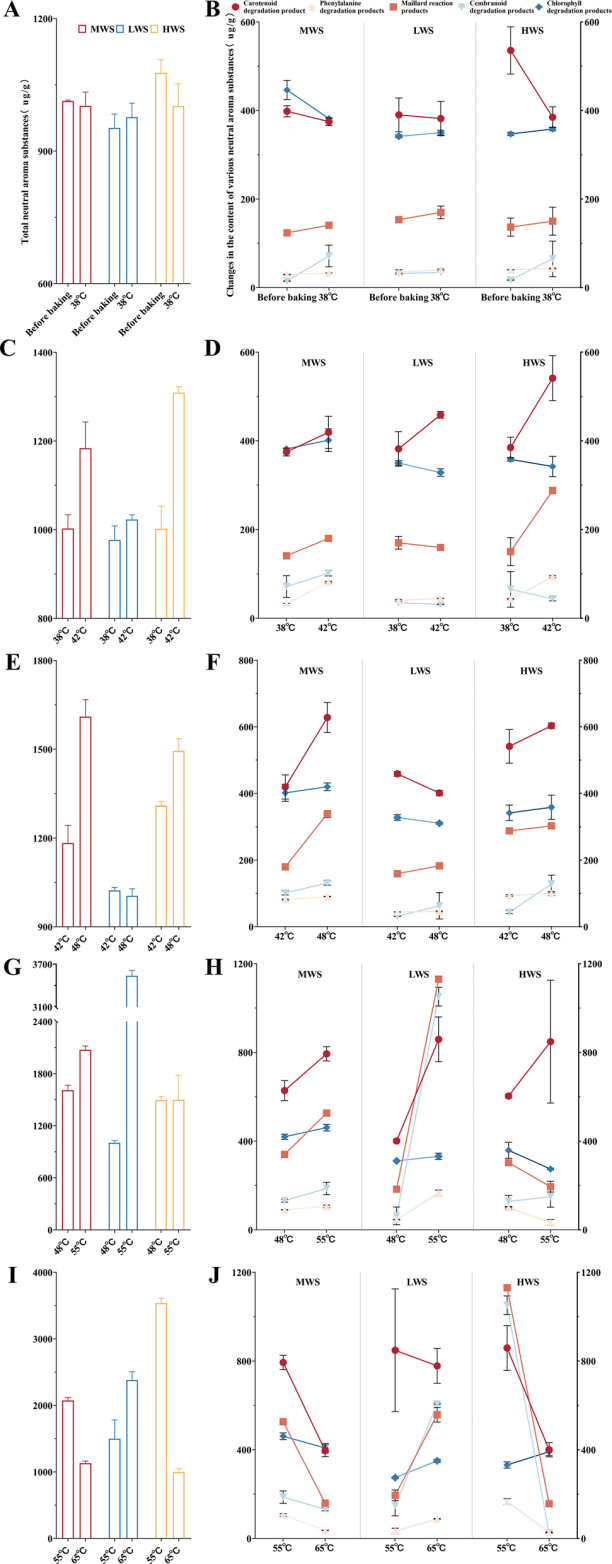
Adjust the fan speed of each baking stage in turn to obtain a set of better fan parameters. (**A**) Before the dry bulb temperature of 38 °C, the change trend of the total amount of neutral aroma substances in different treatments. (**B**) Before the dry bulb temperature of 38 °C, the change trend of various neutral aroma substances in different treatments. (**C, D**) are the 38–42 °C stage. (**E**, **F**) are 42–48 °C stage. (**G**, **H**) are the 48–55 °C stage. (**I, J**) are the 55–65 °C stage. (Images by GraphPad Prism8).

Up to the dry bulb temperature of 38 °C, only the total amount of neutral aroma substances in the LWS treatment showed an upward trend (Fig. 1A). In the three groups of treatments, the content of carotenoid degradation products in LWS decreased the least, and the content of Maillard reaction products increased the most. The content of chlorophyll degradation products increased the most in the HWS, and was 0.86% higher than that in the LWS. The content growth trend of the cembranoid degradation products was the slowest in the LWS and the fastest in the MWS. The content of phenylalanine degradation products in the three groups did not change significantly (Fig. 1B). It can be concluded that up to 38 °C, the use of lower wind speed can effectively increase the content of neutral aroma substances in flue-cured tobacco, and the wind speed is optimal at 750 r/min.

Based on the previous stage experiment, the wind speed of the 38–42 °C stage was screened. The total amount of neutral aroma substances in the three groups increased: MWS increased by 18.07%, LWS increased by 4.7%, and HWS increased by 30.68% (Fig. 1C). Among them, the content of all kinds of neutral aroma components in MWS showed an upward trend, and the degradation products of phenylalanine increased most in MWS. Although the content of chlorophyll degradation products and cembranoid degradation products of HWS decreased, the growth trend of carotenoid degradation products and Maillard reaction products of HWS was faster than that of MWS. The contents of chlorophyll degradation products, cembranoid degradation products and Maillard reaction products of LWS decreased, and the content of phenylalanine degradation products changed little (Fig. 1D). Therefore, it is more appropriate to use high wind speed in the 38–42 °C stage, and the fan speed should be maintained at 900 r/min.

According to the results of the previous stage, the wind speed in the 42–48 °C stage was screened. The total amount of neutral aroma substances in LWS decreased, the total amount of neutral aroma substances in MWS and HWS increased, and MWS increased the most, by 36% (Fig. 1E). Among them, LWS only increased the content of cembranoid degradation products and Maillard reaction products. The contents of neutral aroma components in MWS and HWS increased. The growth rates of carotenoid degradation products, Maillard reaction products and phenylalanine degradation products in MWS were 38.37%, 83.73% and 4.77% higher than those in HWS, respectively. The growth rate of cembranoid degradation products and chlorophyll degradation products was the highest in HWS (Fig. 1F). It is evident that, at the stage of 42–48 °C, it is still necessary to maintain a high wind speed, but a slight reduction in the fan speed can further transform the aroma substances of flue-cured tobacco. Therefore, the optimal fan speed was determined to be 1350 r/min at this stage.

Based on the results of the previous stage test, the parameters of the fan at 48–55 °C were screened. The total amount of neutral aroma substances in MWS and LWS increased by 28.83% and 252.76%(2533.81 ug/g) , respectively, while the total amount of neutral aroma substances in HWS remained basically unchanged (Fig. 1G). Among them, except for carotenoid degradation products and cembranoid degradation products, the other neutral aroma components of HWS decreased significantly, and the contents of all kinds of neutral aroma components of MWS and LWS increased. The upward trend of various neutral aroma substances in LWS was more obvious than that in MWS, and the growth rate was greater (Fig. 1H). Therefore, in the 48–55 °C stage, the wind speed inside the barn should begin to decrease, so maintaining the fan speed at 850 r/min is more conducive to the fixation of aroma substances in flue-cured tobacco.

According to the results of the previous stage test, the wind speed of the 55–65 °C stage was screened. The total amount of neutral aroma substances in MWS and HWS decreased significantly, and the content of neutral aroma substances in LWS increased by 58.83% (Fig. 1I). Among them, the content of all neutral aroma substances in MWS decreased slightly, while the content of chlorophyll degradation products in HWS increased, but the content of other kinds of neutral aroma substances decreased greatly. The degradation products of LWS carotenoids decreased by 8.78%, and other neutral aroma substances increased significantly (Fig. 1J). It is evident that in the final stage of baking, a decrease in wind speed is beneficial to the further fixation of aroma substances, so it is appropriate to maintain the fan speed at 700 r/min.

**Figure 2:**
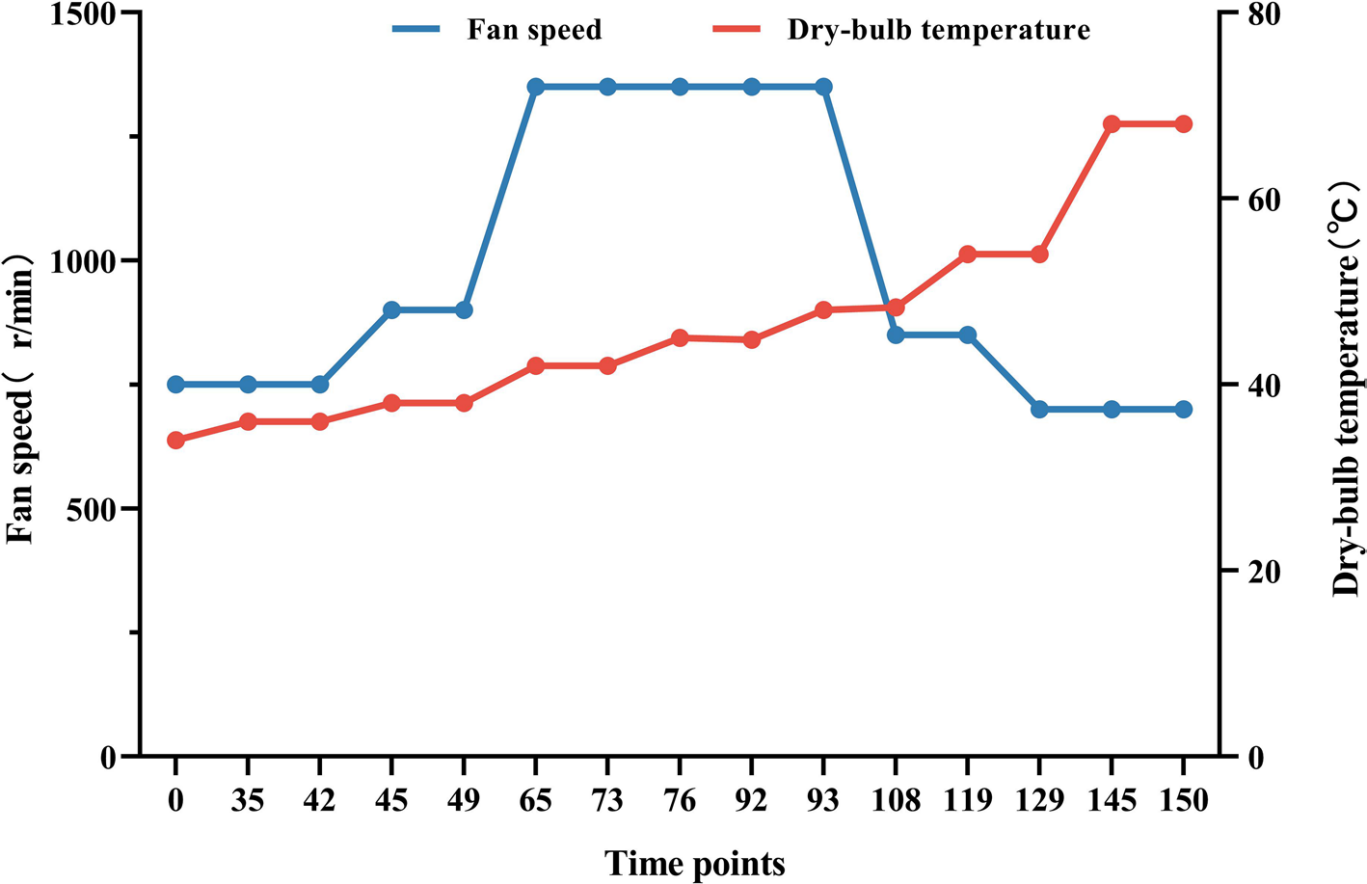
Optimized fan parameters (Images by GraphPad Prism8).

According to the test results of each stage, the optimized fan parameters were obtained, as shown in Fig. 2.

### Effect of fan parameter adjustment on the carbohydrate content of flue-cured tobacco leaves

**Figure 3:**
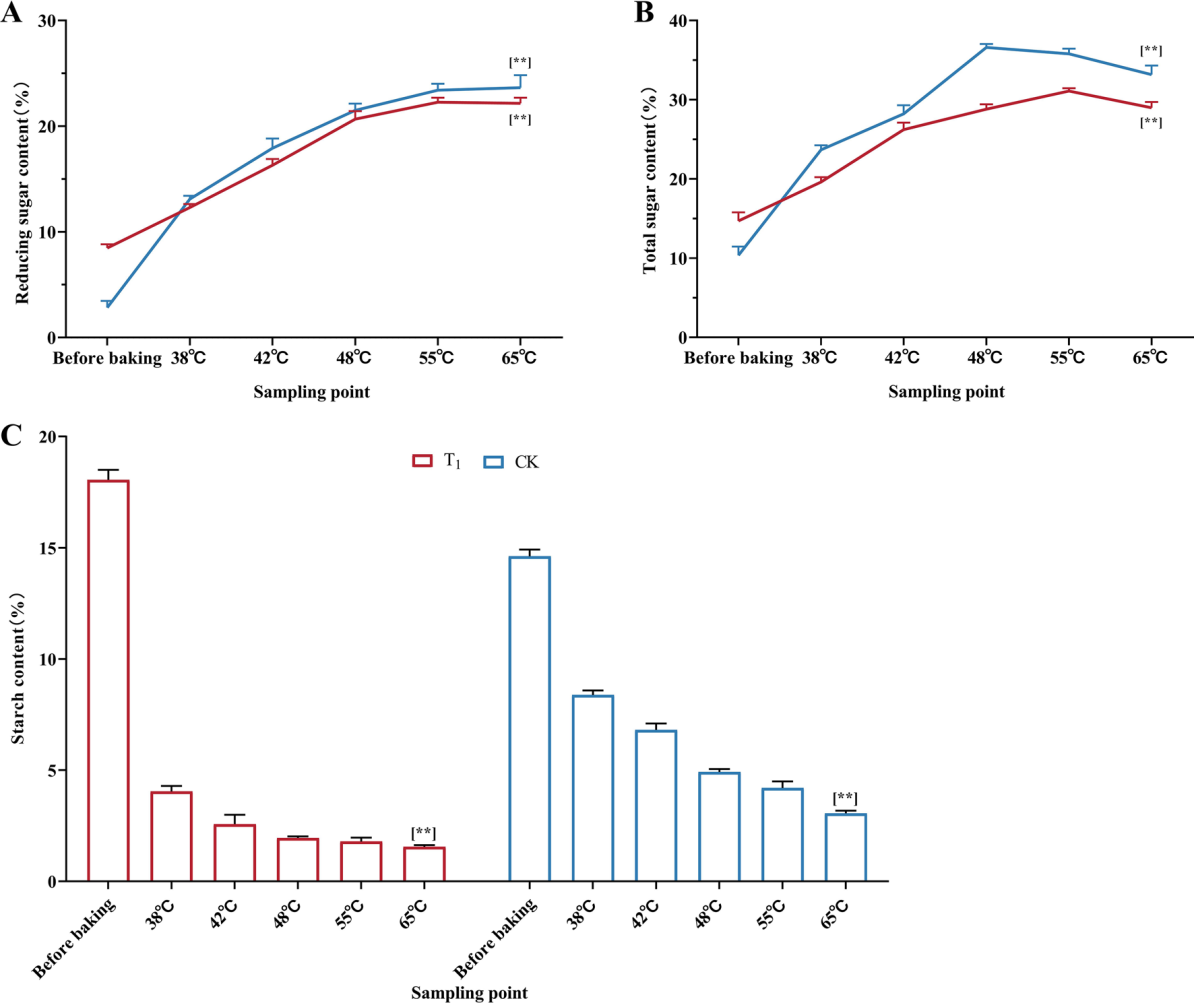
Taking the baking process before optimization (CK) as the control, the changes in carbohydrate content in tobacco leaves during the baking process of the optimized baking process (T_1_) were studied. (**A**) The change in reducing sugar content in the baking process. (**B**) The change in total sugar content in the baking process. (**C**) The change trend of starch content during baking. **, **** represents a significant difference when *p* < 0.05, *p* < 0.01. (Images by GraphPad Prism8).

The two treatments were consistent in the trend of reducing sugar (Fig. 3A) and total sugar content (Fig. 3B). Although the content of two kinds of sugar in CK treatment was significantly higher than that in T_1_ treatment, the sugar content of CK was too high, which would have a negative impact on the sensory quality of tobacco leaves. The content of two kinds of sugar in T_1_ treatment was within the range of high quality tobacco leaves (Reducing sugar: 22.25%,Total sugar: 29.20%).

Figure 3C shows that the starch content of tobacco leaves in both treatments exhibited a downward trend during the curing process. The starch content of fresh tobacco leaves treated with T_1_ was higher than that of CK. Compared with CK, the starch content of the T_1_ treatment decreased rapidly, and starch decomposition was greater in the early stage of baking. During the baking process, the starch content of the T_1_ treatment was always lower than that of CK.

### Effect of fan parameter adjustment on aroma substances and petroleum ether extract content of tobacco leaves

**Figure 4:**
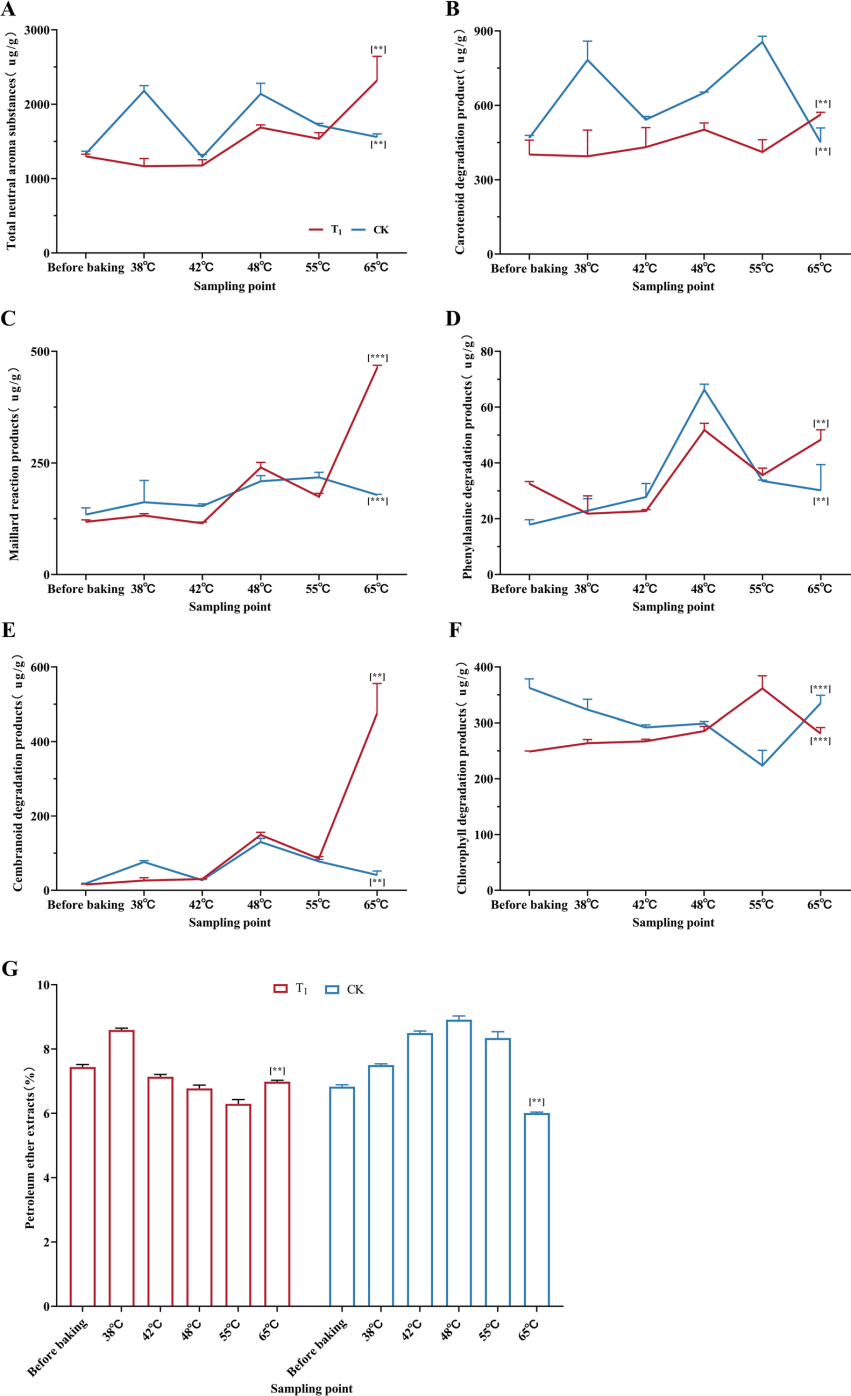
Using the preoptimized baking process (CK) as a control, the changes in neutral aroma substances and petroleum ether extract content in tobacco leaves during the optimized baking process (T_1_) were studied. (**A**) Changes in the total amount of neutral aroma substances during the baking process. (**B**) Changes in the content of carotenoid degradation products during baking. (**C**) Changes in the content of Maillard reaction products during baking. (**D**) Changes in the content of phenylalanine degradation products during the baking process. (**E**) Changes in the content of cembranoid degradation products during baking. (**F**) Changes in the content of chlorophyll degradation products during baking. (**G**) Changes in the content of petroleum ether extract during baking. **, ****Represents a significant difference when *p* < 0.05, *p* < 0.01. (Images by GraphPad Prism8).

From Fig. 4A, it can be seen that the total amount of neutral aroma substances in fresh tobacco leaves of the two treatments is slightly different. Among them, the total amount of neutral aroma substances in the T_1_ treatment showed an overall upward trend and increased significantly from 42 °C to 48 °C and after 55 °C. The total amount of neutral aroma substances in the T_1_ treatment was significantly higher than that in the CK treatment.

From Fig. 4B, it can be seen that the change range of carotenoid degradation product content in the T_1_ treatment is smaller than that in the CK treatment, and there is a trend of decreasing first and then increasing before and after 55 °C. The carotenoid degradation products of flue-cured tobacco leaves treated with T_1_ were 161.07 µg/g higher than those of fresh tobacco leaves. The content of carotenoid degradation products in the T_1_ treatment was significantly higher than that in the CK treatment.

From Fig. 4C, it can be seen that the difference in Maillard reaction product content between the T_1_ treatment and CK treatment was small during the fresh tobacco leaf period. Before 48 °C, the change trend of the two treatments was the same, and the change range of the T_1_ treatment was greater. After 55 °C, the content of the CK treatment decreased, and the content of the T_1_ treatment increased. The content of Maillard reaction products in the T_1_ treatment was significantly higher than that in the CK.

Figure 4D shows that the change trend of phenylalanine degradation products in the two treatments was consistent during the baking process, but the change range of the CK treatment was larger. The changes in the two treatments before 38 °C were opposite, the content of T_1_ decreased, the content of CK treatment increased, and the change trend of the two treatments after 55 °C was opposite. The content of phenylalanine degradation products in cured tobacco leaves of T_1_ was significantly higher than that of CK.

From Fig. 4E, it can be seen that the content of cembranoid degradation products in fresh tobacco leaves of the two treatments is basically the same. CK treatment changed more dramatically before 42 °C. The two treatments were consistent in the middle stage of baking, but the material conversion of T_1_ was more intense. After 55 °C, the content of cembranoid degradation products in the T_1_ treatment increased rapidly. The content of cembranoid degradation products in cured tobacco leaves of T_1_ was significantly higher than that of CK.

From Fig. 4F, it can be seen that the two treatments have the opposite trend in the content of chlorophyll degradation products. The T_1_ treatment increased first and then decreased before and after 55 °C, and the CK treatment decreased first and then increased before and after 55 °C. The content of chlorophyll degradation products in CK was significantly higher than that in T_1_.

It can be seen from Fig. 4G that the content of petroleum ether extract in the T_1_ treatment gradually increased after baking, reached a high point at 38 °C, then gradually decreased, and gradually increased after 55 °C. The content of petroleum ether in the CK treatment increased gradually after baking, reached a high point at 48 °C, and then decreased gradually. The content of petroleum ether extract in T_1_ was significantly higher than that in CK.

### Effects of fan parameters on metabolites of flue-cured tobacco after adjustment

**Figure 5:**
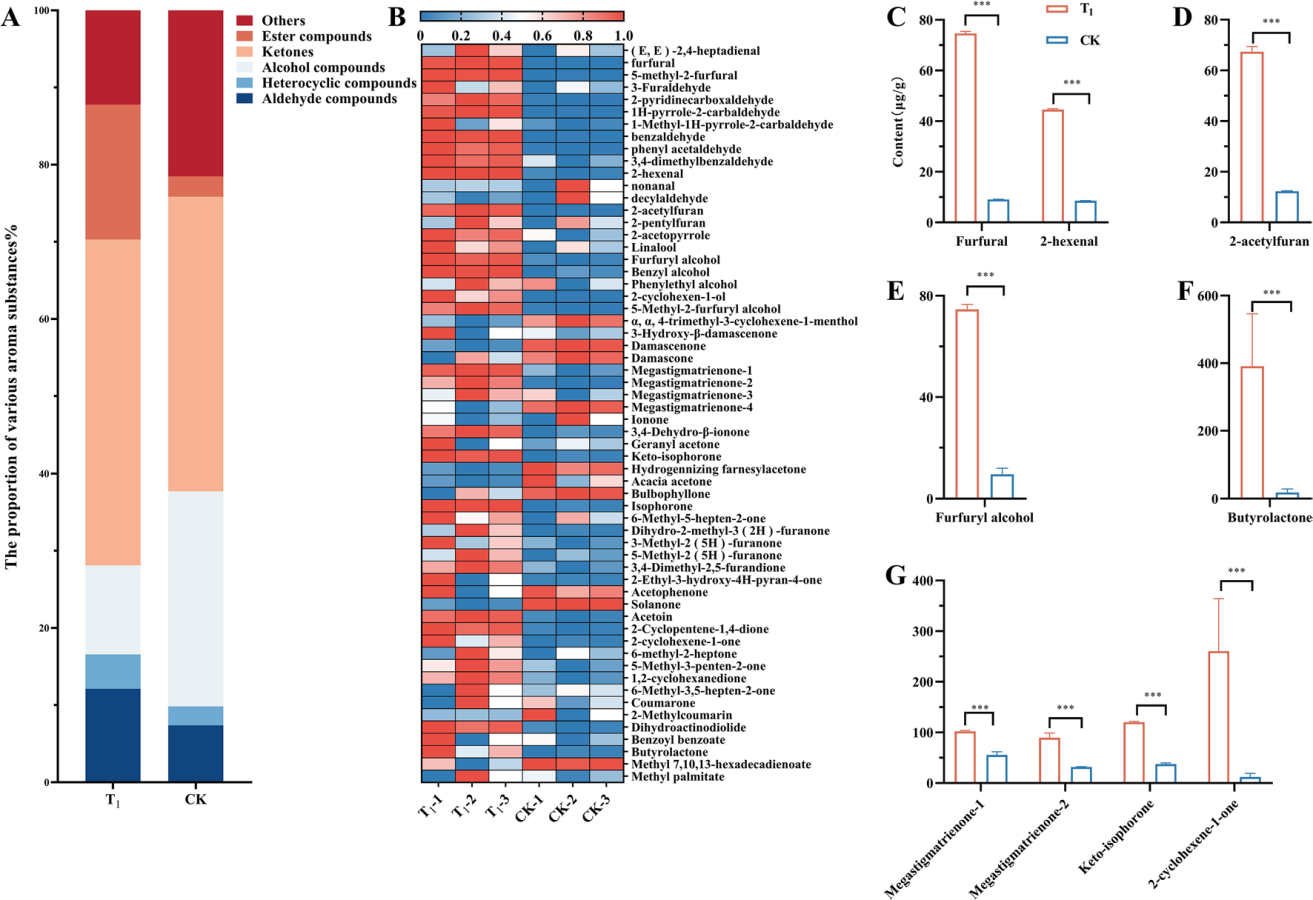
Using the preoptimized baking process (CK) as a control, the metabolic changes in aromatic substances in tobacco leaves after the optimized baking process (T_1_) were studied. (**A**) The content of various aroma components. (**B**) Heatmap based on aroma metabolites. (C-G) Extremely significant differences in various types of metabolites. **, **** Represents a significant difference when *p* < 0.05, *p* < 0.01. (Images by GraphPad Prism8).

To more intuitively evaluate the effect of the baking process on tobacco metabolism after optimizing fan parameters, the contents of metabolites in tobacco leaves before and after optimization were measured. Through targeted metabolomics analysis, alcohols, esters, heterocycles, aldehydes, ketones and other compounds in tobacco leaves were measured, and a total of 60 products related to aroma substance metabolism were also measured. The contents of aldehydes, heterocyclic compounds, ketones and esters in T_1_ were higher than those in CK (Fig. 5A). The content of most aroma metabolites in the T_1_ treatment was higher than that in the CK (Fig. 5B). After the significant analysis of the content of each metabolite, it was found that the content of furfural and 2-hexenal in aldehydes (Fig. 5C), 2-acetylfuran in heterocyclic compounds (Fig. 5D), furfuryl alcohol in alcohols (Fig. 5E), butyrolactone in esters (Fig. 5F), and megastigmatrienone-1, megastigmatrienone-2, isophorone oxide and 2-cyclohexene-1-one in ketones (Fig. 5G) in T_1_ were significantly higher than those in CK.

## Discussion

The aroma of tobacco leaves is an important evaluation standard of their quality^33,34^, and depends on the accumulation of a large number of chemical components, the most important of which are aroma substances represented by Maillard reaction products and carotenoid degradation products^35^. The accumulation of aroma substances has certain rules^36,37^. High molecular weight substances break bonds and degrade during the yellowing period of tobacco leaves, and there are many aroma precursors in the products. Therefore, low wind speed is used during the yellowing period of tobacco leaves to maintain high moisture and high respiration efficiency in the leaves, which is conducive to promoting the degradation of macromolecular substances^38^. Similarly, in the color fixing period of tobacco leaves, the conversion of aroma substances is a more active stage. However, unlike the yellowing period, maintaining excessive moisture during the color fixing period leads to enzymatic reaction of tobacco leaves, browning of tobacco leaves, and a large loss of nutrients. Therefore, high wind speed should be maintained to quickly reduce moisture content within the tobacco leaves. During the drying period of tobacco leaves, the temperature rises, the chemical reaction of the leaves basically stops, and the aroma substances are easily volatilized and lost. The general trend of the change of the test results and the theory of others can be mutually verified, which proves that the test results are very scientific.

However, there are some differences between this study and previous studies. In the previous study, the aroma substances of tobacco leaves in the later stage of curing will gradually lose with the increase of time.After optimizing the fan parameters, the most intuitive change in the accumulation trend of aroma substances in tobacco leaves was that the content of aroma substances reached a high point from 48 °C before optimization (CK), then decreased, and became optimized (T_1_). The content of aroma substances rose again after 55 °C, which may have been because after improving the fan parameters, the control of the water loss rate of tobacco leaves was more scientific, the tobacco leaves underwent severe material transformation during the yellowing period, and the accumulation of aroma substances was greater. However, the color fixing period is still capable of active material transformation, which makes its content decrease at a lower rate; the lower wind speed was used in the drying period, which was more favorable for the solidification of gas matter, so it showed an upward trend after 55 °C. This change better shows that the tobacco material is fully transformed in the early stage of curing, and this change is more conducive to fixing the aroma substances produced by tobacco curing.

Sugar and lipids in tobacco are the main precursors of tobacco aroma^39^. The Maillard reaction and carotenoid degradation are the main sources of tobacco aroma substances, and their reaction degree is deeply affected by carbohydrate content, such as sugar^40^. Therefore, the changes of several carbohydrates that may affect the accumulation of aroma substances were also considered.The change trend of carbohydrates in tobacco leaves verified the above conclusions. Starch and other macromolecular substances are rapidly decomposed in the early stage, and reducing sugar is rapidly accumulated. On the one hand, this provides support for the violent reaction of internal substances in tobacco leaves. On the other hand, sugars are also direct participants in the accumulation of major aroma substances, such as the Maillard reaction^41^.

Petroleum ether extract is an organic mixture, including lipids, sterols, organic acids and other aroma-related substances, and is positively correlated with the quality of tobacco leaves^42,43^. In the verification test, the change trend was basically the same as that of tobacco aroma substances, which also verifies that the optimization of fan parameters is conducive to the accumulation of aroma substances in tobacco leaves.

From the perspective of metabolism, the experimental results showed that after optimizing the fan parameters, the tobacco leaves exhibited significant advantages in the enrichment pathways of aldehydes, alcohols, heterocycles, ketones, and esters. This may have been due to the optimization of the fan parameters, which provide better conditions for the transformation of precursors for the metabolism of aroma substances in tobacco leaves^44,45^.

## Conclusion

In summary, the fan parameters in the baking process were studied to make the wind speed more reasonable and controllable in each stage, which was beneficial to improve the quality of flue-cured tobacco. First of all, it is beneficial to the degradation of macromolecular substances in the early stage of tobacco baking and the conversion into the main precursor substances of aroma substances. Secondly, in the middle stage of curing, it can provide a more suitable environment for the internal material transformation of tobacco leaves ; finally, the aroma substances transformed in the early and middle stages can be better fixed and dry matter loss can be reduced. Therefore, the study of wind speed parameters should be the focus of flue-cured tobacco research.

## Ethics approval

In this study, the collection of plant samples was carried out under the supervision and guidance of the sponsors and relevant departments, and in full compliance with laws and regulations. This study was conducted under legal and compliant conditions.

## Data Availability

Data will be made available on request. Cheng Lin Sun should be contacted if someone wants to request the data from this study.
